# Rabies1Read before the Pathological Society of Rochester, January 17, 1901.

**Published:** 1901-05

**Authors:** Mazyck P. Ravenel

**Affiliations:** Philadelphia, Pa.; Bacteriologist of the State Live Stock Sanitary Board of Pennsylvania


### RABIES.[1]

By MAZYCK P. RAVENEL, M. D., Philadelphia, Pa.,
Bacteriologist of the State Live Stock Sanitary Board of Pennsylvania.

FEW diseases have been known and described for as many centuries as rabies, yet there are perhaps none about which so many misstatements are made, or such erroneous opinions held, not only by the public who are not expected to be students of disease, but also by those whose profession leads them to the investigation of such matters. Some of these false ideas have no doubt come down to us from the days when the origin of all diseases was shrouded in mystery and epidemics were looked upon as the visitations of offended deities. It cannot be denied that rabies has resisted the probe of moden research with great persistence, and on this account perhaps the old notions have clung to it. On the other hand, however, there is too often seen an apparent disposition to deny the most palpable facts concerning it, an attitude which has hindered the spread of knowledge and checked a fair investigation.

More than 300 years before the birth of Christ, Aristotle wrote, "Dogs suffer from madness which puts them in a state of fury, and all the animals they bite when in this condition, become also attacked by rabies;" a statement which shows even at this early date a clear recognition of the communicability of the disease by the bite of the dog. In the first century A.D., Celsius alluded to rabies in man and first employed the term "hydrophobia." From this early period to the present time, with the exception of the middle ages, there has been frequent mention of outbreaks of rabies, and descriptions given which prove that the disease was well known, and in all respects identical with what we are only too familiar at the present day. As early as 1591 the transmission of rabies to man by the bite of wolves is recorded. During the last part of the eighteenth and first part of the nineteenth centuries, rabies spread over the whole of Europe, and numerous epidemics were noted during the century just

---

1. Read before the Pathological Society of Rochester, January 17, 1901.

closed. In the face of this mass of testimony it is amazing that there are still men who profess to doubt the existence of such a disease at all, or if they acknowledge this, deny its transmissibility to man. The ravages of rabies during the last century drew to it the attention of many of the most able men who have adorned the ranks of the medical and veterinary professions. Without detracting from the work of any of these, it is safe to say that to Pasteur we owe the larger part of our exact knowledge regarding the nature of this fearful malady, and to his masterly researches are due the preventive inoculations, which are justly regarded as one of the greatest triumphs of medicine in any age. With the completion of this work our knowledge of rabies was largely extended. We know the clinical picture in man and in many of the lower animals, the symptoms which foretold so surely a prompt and awful death, the distribution of the virus in the system of the affected man or animal, its behavior under many conditions, how its power could be increased or diminished at pleasure, its mode of transmission from animal to animal, and even the method of guarding against infection, yet with all this knowledge we were entirely ignorant of the exact nature of the virus, as indeed we are at the present day, and we knew of no characteristic change in any part of the body which enabled us to say whether or not the animal had died of rabies.

The absence of distinctive post mortem lesions has no doubt given some ground to the skeptics for their attacks on the reality of the disease. As has been well stated by Babès, it would seem "that this disease so clearly characterised by a train of symptoms, constant in their character ought also to present characteristic lesions in the nerve centers, and especially in the ganglia, which preside over the production of symptoms," an opinion which has been generally accepted and acted on by many students, their object being not only to disclose the cause of the striking symptoms, but also to find methods of diagnosis more rapid and more certain than any yet known.

Rabies is an acute infectious disease, due to a specific virus, which is inoculable, and usually transmitted from one animal to another through the saliva. Under what may be called natural conditions, the disease occurs chiefly in the canine race, though all mammifers, including man, and many birds are susceptible to it. Transmission to man is practically always through the bite of a dog or wolf, and more rarely of the fox.

The exact nature of the virus is unknown, but there can be no doubt that it is some organism which has the power of growing and multiplying in the body, and of forming a toxic substance which has a selective or specific action on the central nervous system. It is found most concentrated and purest in the medulla oblongata, though all parts of the brain and cord contain it constantly. It is excreted principally by the parotid gland, but also by the other salivary glands, the lachrymal gland, the pancreas and the mammary glands. The virulence of the saliva may be entirely removed by filtration through porcelain, proving that it is due to some solid substance which the filter retains. The virus is never found in the blood, liver, spleen or kidneys. Absorption of the virus through unabraded mucous surfaces is said to occur in rare instances. It has been proven by Nocard and Roux that the virus may be contained in the saliva, at least three days before the animal shows any signs of illness, and it is known also that even the strongest virus remains in the central nervous system for some time before producing recognisable symptoms. Experimentally, the disease is reproduced most certainly and with the greatest certainty by the injection of a portion of the medulla under the dura mater of the brain, but it follows almost as surely when the injection is made into the anterior chamber of the eye or into one of the large nerve trunks, though not so rapidly. Subcutaneous inoculation cannot be relied upon, as the nerves are not always injured, but inoculation into the substance of a muscle seldom fails to produce the disease, especially if the part has a rich supply of nerves. Inoculation into the circulation succeeds at times in small animals, but not in large ones like the horse or cow.

In the vast majority of cases, both in man and in animals, rabies is produced by direct inoculation of the virus contained in the saliva of a rabid animal, the introduction being effected through wounds inflicted by the teeth. Having gained entrance through the skin the virus may remain for a long time at the site of the injury. It travels along the nerve tracts towards the central nervous system where it remains for a time, no doubt increasing in quantity before the typical symptoms are produced. This mode of transmission along the nerves can be proven in several ways. When the inoculation has been made in the sciatic nerve, for instance, the invasion of the brain and medulla can be prevented by section of the cord. Also, if we inoculate an animal in the leg, and kill it after a time, but before symptoms of rabies appear, the nerves of the leg will be found to contain the virus, and often also the portion of the cord which receives the nerves, while the brain and rest of the cord are free from it. The circulation and lymphatic system probably at times take part in the absorption and distribution of the virus also.

The danger of inoculation after the bite of a rabid animal, as well as the time which may elapse between the injury and the appearance of symptoms, is determined by (1) the seat and extent of the injury, and (2) the quantity and strength of the virus. Deep and lacerated wounds about the face and hands where the nerves are abundant are particularly dangerous, and the period of incubation short, while slight wounds, made in portions of the body covered by clothing, or where the skin is thick may be followed by no ill-effect, or else only after a long period of incubation. Among the lower animals it is known that dogs with long hair, such as the poodle and spaniel, are less often affected than short haired species, and that sheep are more liable to contagion when shorn. The protective influence of clothing may be shown experimentally by exposing rabbits to the bite of a mad dog, some shaven and some with fur intact. A large proportion of those bitten through the fur will escape, even when the teeth have passed well into the skin. The saliva with its contained virus is wiped off by the clothing or fur in these cases. The bite of carnivorous animals is much more dangerous than that of herbivorous, and the bite of wolves is especially fatal. This is due probably in part to their savage attack, inflicting extensive wounds about the face, and in part to a greater strength of the virus, as found in this species. Thus the former mortality from the bite of wolves is placed at 40 to 60 per cent., while that from the bite of dogs is only 16 per cent. The Pasteur preventive treatment is also said to be somewhat less effective against the bites of these animals.

*Post Mortem Diagnosis.* — As has been said, there are no anatomical changes which enable us to make a post mortem diagnosis with any degree of certainty. The presence of foreign bodies, such as pebbles, wood, air, straw, etc., in the stomach, and the absence of food, is indicative of death from rabies, but the only sure means of diagnosis heretofore has been the inoculation of animals with a portion of the brain or spinal cord of the suspected dog, a procedure which is often attended with difficulty on account of the poor condition in which much material is collected.

It is needless to dwell on the anxiety of the family of a person who has been bitten by a dog suspected of rabies, and of the patient himself, while awaiting the tardy verdict of inoculation; or on the value of the time that may be lost in instituting preventive treatment. It is hard to overestimate the importance of a simple and positive method of rapid diagnosis.

A short review of the work done by different observers with this object may be of interest:

The first step in this direction seems to have been due to Pollailon and Nepveu, who described the lesions observed in a man who had died of rabies. They noted that the whole cerebrospinal axis was strongly congested, and that the ganglion of Gasser was hyperemic and infiltrated with round or oval cells, some of them being hyaline in appearance, and which they considered "probably epithelioid cells from the capsule of the ganglion cells." The work of Balzer, calling attention to the changes in the central nervous system, was followed in 1874 and 1875 by Benedikt's article, both authors described the distention of vessels in the nervous centers, accompanied by an extravasation of erythrocytes and leukocytes into the perivascular spaces. Kolesnikoff, in 1875, described besides an invasion of the pericellular spaces by round cells occurring in the hemisphere, cerebellum, spinal cord, and the sympathetic and intervertebral ganglia. Schaffer also made a careful study of the vascular and cellular changes found in the case of a man, and called attention to a hyaline and fibrillar degeneration and vacuolation of the cells of the anterior horns of the cord. In 1886, Babès concluded as a result of numerous researches, made in man and in dogs, both natural and experimental cases, that the essential lesion of rabies consisted in an accumulation of embryonic cells in the neighborhood of the central canal, and especially about the large modified cells of the motor centers of the bulb and cord. Writing again in 1892, Babès reaffirmed his observations and insisted on the profound changes taking place in the nerve cells. He held that it was possible to make a rapid diagnosis of the disease by a microscopic examination of the bulb and cord. He described in the bulb what he considered the diagnostic lesion of the disease—namely, the pericellular accumulations of embryonal cells described by Kolesnikoff, and for which he proposed the name "rabic tubercle." The cells of the bulbar nuclei undergo degeneration and present the various stages of chromotolysis. There is loss of the prolongations and a progressive modification and even total disappearance of the nuclei, a dilatation of the pericellular space, and an invasion not only of this space but also of the nerve cells by embryonal cells, and at the same time small corpuscles which are hyaline, brownish, and in parts metachromatic. Many of the nerve cells become surrounded by a large zone of embryonal cells, and when the cell is completely degenerated these occupy the cell area and constitute the rabic tubercle.

Since 1891 Babès has examined the bulb of 487 dogs, controlling his results always by inoculation of rabbits. His results have led him to trust this means of diagnosis and he still holds it to be one of the best means of rapid diagnosis at our disposal, although except in Babès own laboratory the method does not seem to have been practiced, at which he expresses a surprise, which appears justifiable. Recently Nélis, working with Van Gehuchten, discovered in the spinal ganglia of two men who had died of rabies, and of a number of animals, peculiar changes which they considered to be the diagnostic lesion of the disease. They have confirmed all the lesions described by other authors, but in addition have noted what they considered to be more diagnostic than any other. The most profound, the most constant, and the earliest lesions are noted in the peripheral, cerebral and sympathetic ganglia, and the changes are especially marked in the intervertebral ganglia, and in the plexiform ganglia of the pneumogastric nerve. Normally, these ganglia are composed of a supporting tissue holding in its meshes the nerve cells, each one of which is enclosed in an endothelial capsule. The changes characteristic of rabies consist in the atrophy, the invasion, and the destruction of the nerve cells, brought about by new formed cells derived from the capsule, which appear between the cell body and its endothelial capsule. These new-formed cells increase in number, invade the protoplasm of the nerve cell, and finally completely occupy the entire capsule. These changes are widespread, but few capsules remain unaffected, and in advanced cases the section has very much the appearance of an alveolar sarcoma. The changes vary in intensity in different animals. They are most marked in the dog, less in man, and less still in rabbits. In dogs and rabbits the changes are more marked in the cerebral than in the spinal ganglion.

The authors have considered in detail the findings of previous workers, but without exception they consider them as secondary and without any importance, either in the production of symptoms or in making a diagnosis. They hold that Babès has attached an undue importance to his rabic tubercle. In their first publication they held that "these lesions are specific of the rabic infection, if not by their nature, at least by their localisation." In a later note, in answer to objections raised by Nocard and others, Van Gehuchten says that "the lesions of the cerebrospinal and sympathetic ganglia which he and Nélis have discovered are not specific of rabies in general; they are only specific of the disease as it occurs naturally." It is to be noted that the authors have made no claim regarding an early diagnosis of rabies, but only a rapid method. Van Gehuchten says his method "concerns not a precocious diagnosis, but only a rapid diagnosis in rabies." This has been further shown by the experiments of Cuillé and Vallée, who, after inoculating dogs with the virus of rabies, sacrificed them at various times after the appearance of symptoms. They found that in animals in which the disease had just become manifest the lesions in the plexiform ganglion were slight or absent. They conclude that as a means of diagnosis the method of Van Gehuchten and Nélis has great value in the case of animals in which the disease has run its full course, ending in death.

The subject has been studied also by numerous veterinarians of note in Europe, notably Dégive and Hebrandt of the State School at Cureghem, and Nocard and Vallée of France, all of whom have obtained results confirming the value of the discovery. Quite recently, Crocq has made a most thorough and complete review of the literature on the subject and a study of the method. He believes that Van Gehuchten is mistaken in considering the perivascular and pericellular neo-formations described by Kolesnikoff and Babès as unimportant changes and believes that by their constant character, their localisation and evolution they have a considerable diagnostic value. He says: "The lesions described by Babès, and Van Gehuchten and Nélis, are both remarkable. They are so great and clearly defined that I can understand how the authors each on his side may have concluded that the alterations described by them were specific." The procedure of Van Gehuchten and Nélis is, however, more simple and rapid than the method of Babès, and on this account is to be preferred.

The method of procedure recommended by Van Gehuchten and Nélis is as follows: The ganglion is put at once into absolute alcohol, in which it is left for twelve hours, the alcohol being changed once. It is then transferred for one hour to a mixture of absolute alcohol and chloroform; next put for one hour in pure chloroform; then for one hour in a mixture of chloroform and paraffin, and lastly in pure paraffin for one hour. The sections are put in the oven for a few minutes, then passed through xylol, absolute alcohol and 90 per cent. alcohol, after which they are stained for five minutes in methylene blue, according to Nissl's formula, differentiated in 90 per cent. alcohol, dehydrated in absolute alcohol, and cleared in essence of cajuput and xylol. If frozen sections are cut, they are put for a few minutes in 90 per cent. or 94 per cent. alcohol.

Realising the great importance of the discovery of Van Gehuchten and Nélis, in conjunction with Dr. D. J. McCarthy, of the William Pepper Laboratory of Clinical Medicine, I undertook in last May a series of experiments to determine its applicability to the examination of the animals suspected of rabies which are frequently sent to the laboratory of the State Live Stock Sanitary Board for diagnosis. In our work we have generally used 10 per cent. formalin for fixing our tissues. They are then transferred to 95 per cent. alcohol, and finally to absolute alcohol. For the most part the tissues have been cut without imbedding, being attached to blocks by the aid of mucilage of gum arabic, though in some cases celloidin has been used. The whole process can be completed in six hours. For the bringing out of the chromatolytic changes the Nissl method has proved the best, but the capsular changes were best brought out in sections stained by hematoxylin and eosin. Since these latter changes are the most essential diagnostic features in the sections, we would suggest that material unfit for the Nissl method will still show the capsular changes when stained by hematoxylin or hemalum and eosin.

Up to the present time we have examined twenty-eight cases of rabies, including eleven dogs suffering with street rabies, one cow, one horse and fifteen rabbits, which were inoculated from these animals, a few being of the second generation, *i.e.*, inoculated from the first experimental animal. In all of the cases studied (28), except that of the horse, suspected of rabies, we obtained positive results in the plexiform ganglia. In one case, however, the changes were slight, and more marked in the peripheral (distal) portion of the ganglion. In twenty-one cases the bulb was examined for the rabic tubercle of Babès. Positive results were obtained in nineteen, though in two cases only chromatolysis, without distinct tubercle formation, was present. It will be seen that in two cases the rabic tubercle of Babès was not found, while the lesions described by Van Gehuchten and Nélis were present, though in one of these the changes in the ganglion were so slight as to leave us in some doubt. In this case, however, the subdural inoculation of rabbits gave positive results, and the microscopic examination of these rabbits showed the bulbar lesions of Babès as well as those of Van Gehuchten and Nélis in the ganglia. In the two cases mentioned in which the bulb showed only chromatolysis without distinct tubercle formation, the lesions in the ganglia were marked. One of these was the cow, which had shown clinical symptoms leading the veterinarian in attendance to suspect rabies. Two rabbits inoculated subdurally with the brain of this cow both died with typical signs of rabies, and by microscopic examination both the rabic tubercle of Babès and the lesions of Van Gehuchten and Nélis were found. These results lead us to believe that the changes in the intervertebral ganglia are more constant than those in the bulb.

Of the rabbits examined three had been inoculated with virus of the second generation from dogs having street rabies. One of these rabbits showed symptoms of the disease on the eleventh day after inoculation, the shortest time which elapsed in any of our cases between inoculation and the appearance of rabiform symptoms. The microscopic examination of these rabbits gave positive results.

Of the dogs examined six had shown unmistakable clinical signs of rabies, while five were only strongly suspected of the disease. All the cases were controlled by the inoculation of rabbits, except two dogs, in which the clinical symptoms left no doubt as to the diagnosis.

Our work has led us also to the study of a number of control cases which are as yet incomplete. So far as we have gone, however, they induce us to believe that the lesions described by Van Gehuchten and Nélis may, for all practical purposes, be regarded as specific of rabies. We bear in mind the opinion of Van Gehuchten himself, and held also by some other observers, that in dogs at least the changes in the cerebro-spinal and sympathetic ganglia are only specific of the natural or street rabies. The lesions produced by the experimental inoculation of the "fixed" virus are not as marked as those in natural cases, and at times may be completely absent. We have not experimented with a fixed virus, but in rabbits inoculated with street virus of the first and second generations the gangliar changes have been marked. We have been convinced that the discovery of Van Gehuchten and Nélis is of great importance from a scientific as well as a humanitarian standpoint. It can be rapidly and easily carried out, and should be a matter of routine in the examination of suspected cases of rabies in every laboratory.

In the examination of the medulla several sections were taken at random for mounting, but serial sections, extensive investigations of different portions of the bulb were not made, inasmuch as we were investigating rapid methods of diagnosis only, and not the essential nature nor the presence of these tubercles in the central nervous system. It is with this understanding that we recommend the examination of the ganglia rather than the bulb.

The attention which the subject has attracted, and the large number of investigations which are being made in consequence, will probably lead to the discovery of isolated cases here and there, in which lesions closely resembling, or perhaps identical with, those under discussion will be found. So far only four such cases have been reported, all in man. While these are sufficient to make us doubt the absolute specificity of the lesion, it can be said with certainty that neither in man nor in any of the lower animals is there any condition known in which these changes appear constantly, except rabies. Their occurrence in this disease is so constant and so marked that we cannot but believe that they have great diagnostic value.

We have as yet failed to find a case in which these changes were present that did not prove to be rabies on inoculation. On the other hand, though we have examined the ganglia of a number of animals, some of which showed a progressive paralysis not unlike that seen in rabies, we have never seen any changes which could be mistaken for these. In a recent publication Hebrant makes a similar statement, mentioning as diseases in which he has made the examination, distemper, cerebellar paralysis, encephlo-myelitis and lead poisoning, all in dogs.

Beyond the diagnostic value of this discovery we feel that it has another and scarcely less important bearing, in that we can say to those who have questioned the reality of this disease as a distinct pathological entity, that there is now an easily demonstrable lesion distinctive of rabies. With this step accomplished, we can expect further advances in the immediate future, and hope that it will not be long before coroners will admit the diagnosis to their records without hesitation, and that health officers will be given full power to impose such regulations as are necessary for the suppression—and let us trust—the eradication of this fearful disease.

It must be remembered when employing this rapid method of diagnosis that while the presence of the changes described in the ganglia enables us to say with great certainty that the animal furnishing them was rabid, the absence of them does not permit us to exclude positively this diagnosis. To obtain the surest results the animal must not be destroyed, but allowed to die. Owing to the quick death which is the rule in rabies this does not entail much loss of time in beginning treatment.

From our study of this subject the following conclusions seem justified:

1. When present, the capsular and cellular changes in the intervertebral ganglia, taken in connection with the clinical manifestations, afford a rapid and trustworthy means of diagnosis of rabies.

2. That when these changes are not present it does not necessarily imply that rabies is not present. The lesions afford contributory evidence more or less valuable, depending on the duration of the clinical manifestations.

3. That in certain cases when the capsular changes are slight, such as in animals dying or killed in the early stages of the disease, the changes are more marked in the distal-peripheral end of the ganglion.

4. That the rabic tubercle of Babès is present sufficiently often to furnish valuable assistance in cases where only the central nervous system is obtainable without any of the ganglia, but in cases where the ganglia can be had they offer a simpler and easier method of diagnosis than do the brain or cord themselves.—*From the Laboratory of the State Live Stock Sanitary Board.*

